# Quantifying robustness of CT-ventilation biomarkers to image noise

**DOI:** 10.3389/fphys.2023.1040028

**Published:** 2023-02-14

**Authors:** Mattison J. Flakus, Antonia E. Wuschner, Eric M. Wallat, Wei Shao, Dhanansayan Shanmuganayagam, Gary E. Christensen, Joseph M. Reinhardt, Ke Li, John E. Bayouth

**Affiliations:** ^1^ Department of Medical Physics, University of Wisconsin-Madison, Madison, WI, United States; ^2^ Department of Medicine, University of Florida, Gainesville, FL, United States; ^3^ Department of Animal Sciences, University of Wisconsin-Madison, Madison, WI, United States; ^4^ Department of Electrical and Computer Engineering, University of Iowa, Iowa City, IA, United States; ^5^ Roy J Carver Department of Biomedical Engineering, University of Iowa, Iowa City, IA, United States; ^6^ Department of Human Oncology, University of Wisconsin-Madison, Madison, WI, United States

**Keywords:** CT, lungs, ventilation, noise, biomarker, registration, 4DCT, Jacobian

## Abstract

**Purpose:** To quantify the impact of image noise on CT-based lung ventilation biomarkers calculated using Jacobian determinant techniques.

**Methods:** Five mechanically ventilated swine were imaged on a multi-row CT scanner with acquisition parameters of 120 kVp and 0.6 mm slice thickness in static and 4-dimensional CT (4DCT) modes with respective pitches of 1 and 0.09. A range of tube current time product (mAs) values were used to vary image dose. On two dates, subjects received two 4DCTs: one with 10 mAs/rotation (low-dose, high-noise) and one with CT simulation standard of care 100 mAs/rotation (high-dose, low-noise). Additionally, 10 intermediate noise level breath-hold (BHCT) scans were acquired with inspiratory and expiratory lung volumes. Images were reconstructed with and without iterative reconstruction (IR) using 1 mm slice thickness. The Jacobian determinant of an estimated transformation from a B-spline deformable image registration was used to create CT-ventilation biomarkers estimating lung tissue expansion. 24 CT-ventilation maps were generated per subject per scan date: four 4DCT ventilation maps (two noise levels each with and without IR) and 20 BHCT ventilation maps (10 noise levels each with and without IR). Biomarkers derived from reduced dose scans were registered to the reference full dose scan for comparison. Evaluation metrics were gamma pass rate (Γ) with 2 mm distance-to-agreement and 6% intensity criterion, voxel-wise Spearman correlation (*ρ*) and Jacobian ratio coefficient of variation (*CoV*_*JR*_).

**Results:** Comparing biomarkers derived from low (CTDI_*vol*_ = 6.07 mGy) and high (CTDI_*vol*_ = 60.7 mGy) dose 4DCT scans, mean Γ, *ρ* and *CoV*_*JR*_ values were 93% ± 3%, 0.88 ± 0.03 and 0.04 ± 0.009, respectively. With IR applied, those values were 93% ± 4%, 0.90 ± 0.04 and 0.03 ± 0.003. Similarly, comparisons between BHCT-based biomarkers with variable dose (CTDI_*vol*_ = 1.35–7.95 mGy) had mean Γ, *ρ* and *CoV*_*JR*_ of 93% ± 4%, 0.97 ± 0.02 and 0.03 ± 0.006 without IR and 93% ± 4%, 0.97 ± 0.03 and 0.03 ± 0.007 with IR. Applying IR did not significantly change any metrics (*p* > 0.05).

**Discussion:** This work demonstrated that CT-ventilation, calculated using the Jacobian determinant of an estimated transformation from a B-spline deformable image registration, is invariant to Hounsfield Unit (HU) variation caused by image noise. This advantageous finding may be leveraged clinically with potential applications including dose reduction and/or acquiring repeated low-dose acquisitions for improved ventilation characterization.

## 1 Introduction

Functional lung biomarkers identify regional variation in lung function, including ventilation and/or perfusion. Ventilation biomarkers have been generated from several medical imaging modalities, including computed tomography (CT) (Simon, 2000; Guerrero et al., 2005; Reinhardt et al., 2008; Kipritidis et al., 2014; Kipritidis et al., 2016; Eslick et al., 2018; Castillo et al., 2019; Shao et al., 2020; Cazoulat et al., 2021). Several methods to derive ventilation information from CT scans have been developed using multiple image acquisition and post-processing techniques. CT-ventilation biomarkers are commonly derived with patient breathing maneuvers of breath-hold CT (BHCT) or free-breathing through four-dimensional CT (4DCT) acquisition. The two primary post-processing techniques that have been implemented are calculating CT-ventilation directly from Hounsfield Units (HU) (Kipritidis et al., 2016) or using the Jacobian determinant of deformable image registration (Reinhardt et al., 2008; Shao et al., 2020). The various methods for calculating regional ventilation from CT scans have been previously reviewed in detail, including descriptions of their uncertainties, validation results and shortcomings (Vinogradskiy, 2019).

One application of ventilation biomarkers is in functional avoidance radiation therapy (RT) in which dose distributions are optimized to reduce dose to functioning lung. Currently being investigated in multiple clinical trials [NCT0252894225 (Yamamoto et al., 2016), NCT0230870926 (Vinogradskiy et al., 2015), NCT0284356827 (Bayouth, 2016)], the goal of functional avoidance RT is to preserve post-RT lung function and mitigate post-RT toxicities. CT-based ventilation biomarkers are particularly advantageous for applications in RT since CT-simulation is routinely acquired for RT treatment planning (Ettinger et al., 2012) and CT has high spatial and temporal resolution. However, CT-ventilation biomarkers have the disadvantage of associated radiation dose, increasing risk to patients (Bagherzadeh et al., 2018). Since there is a direct tradeoff between image dose and image noise in CT imaging, generating biomarkers minimally impacted by image noise would allow for potential dose reduction, broadening clinical applications of the biomarkers. Understanding the relationship between biomarkers and image noise is also critical for evaluating their robustness.

For the purpose of evaluating the impact of image noise on CT-ventilation biomarkers, mitigating the effect of other contributing uncertainties is critical. Mechanically ventilated non-human subjects offer a precisely controlled environment relative to human patients, as demonstrated in previous work (Du et al., 2012; Du et al., 2013; Mistry et al., 2013). Additionally, performing CT imaging in non-human subjects allows greater latitude for increased imaging dose from repeat imaging. Swine models share similarities in genetics, anatomy and bodily function with humans (Schomberg et al., 2016). The novel Wisconsin Miniature Swine (WMS) breed was developed to model human physiology more accurately than conventional breeds (Schomberg et al., 2016). With similar weight and size to humans, WMS are an ideal model for evaluating the relationship between image noise and derived CT-ventilation values. The purpose of this work is to quantify the impact of image noise on CT-ventilation biomarkers calculated using the Jacobian determinant computed directly from the deformable image registration transformation.

## 2 Materials and methods

### 2.1 CT imaging

All CT scans were acquired on a Siemens SOMATOM Definition Edge CT scanner (Siemens Healthineers, Erlangen, Germany) at the University of Wisconsin-Madison (UW-Madison, Madison, WI). First, repeated CT acquisitions of a uniform phantom were acquired with varying image noise levels. Next, quantified image noise from phantom imaging was used to guide selection of relevant image noise levels for acquiring CT scans in mechanically ventilated WMS.

For phantom and WMS scans, static (for BHCT) and 4DCT scans were acquired with constant scan parameters currently use in standard of care CT-simulation at UW-Madison. The corresponding parameter values are 120 kV, 0.5 s tube rotation time, 76.8 mm beam collimation and 128 detector rows. Image noise was varied by changing only the tube current time product between acquisitions. The tube current is measured in units of milliamps (mA) and tube rotation time is measured in seconds (s), giving their product units of mA×s or mAs. The tube current time product variable, commonly referred to as mAs, has an inverse relationship with image noise [defined by the standard deviation of Hounsfield Units (HU)], denoted in Eq. 1, and is proportional to image dose (Eq. 2).

$$Noise\propto {\left(mAs\right)}^{-0.5}$$ (1)

Dose∝mAs (2)

#### 2.1.1 Uniform phantom scans

**FIGURE 1:**
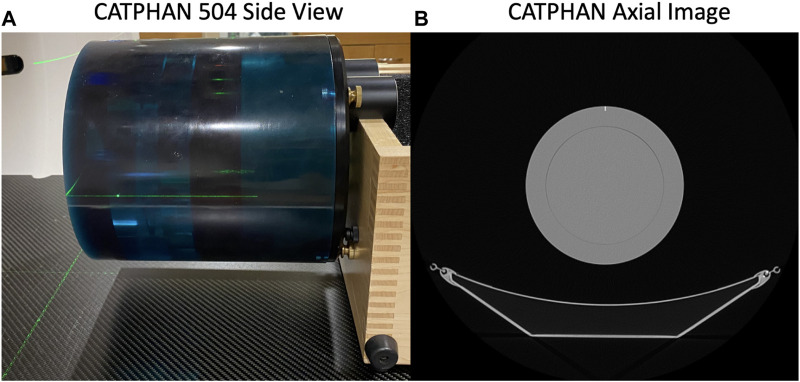
The CATPHAN 504 imaged in this study is shown **(A)** setup on the CT table and **(B)** with an axial CT image of the uniformity region.

The uniformity region (Module CTP486) of a CATPHAN 504 phantom (The Phantom Laboratory, Salem, NY) was imaged in static and 4DCT acquisition modes. Figure 1 shows the phantom setup on the CT table and an axial image of the uniformity region. Repeated helical 4DCT scans with a pitch of 0.09 and static scans with a pitch of 1 were acquired with mAs values ranging from 10 to 100 mAs (corresponding to 20–200 mA with 0.5 s); 10 mAs is the minimum mAs value the scanner allows when a 0.5 s rotation time is used and 100 mAs is the current mAs value used in standard of care CT-simulation 4DCT scans at UW-Madison.

#### 2.1.2 Image noise calculation and mAs parameter selection

In this work, image noise was calculated as the standard deviation of HU in a centrally-placed circular region of interest (ROI) in axial phantom images acquired with each mAs value. All mAs and corresponding image noise values were fit to an exponential using Microsoft Excel (Microsoft, Inc., Redmond, WA) power-law curve-fitting to identify the relationship between image noise and mAs values with constant scan parameters listed previously for both static and 4DCT acquisitions. Equation 3 shows the general relationship between image noise and 4DCT mAs (mAs_4*D*_) with constants *A*_4*D*_ and *B*_4*D*_. Similarly, the relationship between BHCT image noise and mAs (mAs_*BH*_) is given in Eq. 4 (with constants *A*_*BH*_ and *B*_*BH*_). Based on the theoretical relationship between image noise and mAs (Eq. 1), exponential constants *B*_4*D*_ and *B*_*BH*_ are unitless and expected to have numerical values near 0.5. Since noise is expressed in units of HU, *A*_4*D*_ and *A*_*BH*_ have units of HU $\times \sqrt{mAs}$ .

$$Noise_{4DCT}=A_{4D}\times {\left(mAs_{4D}\right)}^{-B_{4D}}\;\;\left[units=HU\right]$$ (3)

$$Noise_{\mathrm{BHCT}}=A_{BH}\times {\left(mAs_{BH}\right)}^{-B_{BH}}\;\;\left[units=HU\right]$$ (4)

At UW-Madison, 4DCTs are currently used for CT-simulation and CT-ventilation calculation. With a low pitch of 0.09, 4DCT scans are time-consuming to acquire and have high associated dose; these aspects limited feasibility of acquiring several consecutive 4DCTs with unique image noise levels. To address this challenge, 4DCTs were acquired at two noise levels and BHCT scans were acquired at multiple intermediate noise levels. With a pitch of 1, BHCT scans require less dose and time than 4DCT acquisitions. Including BHCT imaging has the added benefit of expanding applicability of the study, since BHCT scans are commonly used for CT-ventilation calculation and the further reduced dose broadens potential clinical uses.

To ensure clinically relevant image noise levels were selected for BHCT imaging, mAs_*BH*_ values with image noise at and above current practice (mAs_4*D*_ = 100 mAs/rotation) were chosen. Appropriate mAs_*BH*_ values were determined by setting Eqs 3, 4 equal to discern mAs_*BH*_ with an equivalent noise level to mAs_4*D*_, as listed in Eq. 5. For WMS imaging, 4DCTs were acquired with reduced (10 mAs) and standard of care (100 mAs) mAs values. BHCT images were acquired at intermediate noise levels equivalent to 4DCTs with 15, 20, 25, 30, 35, 40, 60, 70, 80 and 100 mAs according to Eq. 5. Tighter sampling was used at lower equivalent mAs values (15–40 mAs) since the low noise region is the steepest portion of the exponential curve relating image noise and mAs.

$$mAs_{BH}={\left(\frac{A_{BH}}{A_{4D}}\times {\left(mAs_{4D}\right)}^{B_{4D}}\right)}^{\frac{1}{B_{BH}}}$$ (5)

Beyond varying mAs values, application of iterative reconstruction (IR) was used to evaluate the impact of image noise; IR is commonly used for noise reduction. The commercially available Siemens IR algorithm, SAFIRE, was applied with strength three (out of possible strengths 1–5). All 4DCT and BHCT phantom scans used for noise calculation were reconstructed with 512 mm extended field of view (FOV), 1 mm slice thickness, a medium smooth kernel (Br51f) and both with and without SAFIRE3 IR.

#### 2.1.3 Image dose

Image dose for scans at each mAs level was quantified through the CT dose index volume (*CTDI*_*vol*_) for the 32 cm phantom in units of milligray (mGy). For the scan parameters used in this work, 10 and 100 mAs 4DCT scans had corresponding *CTDI*_*vol*_ of 6.07 and 60.7 mGy, respectively. Therefore, 4DCT comparisons between 10 and 100 mAs scans represent differences for dose being reduced by 10 times from current standard of care. Since BHCT acquisitions used a higher pitch, they had reduced dose compared to 4DCTs. As stated in the previous section, BHCT mAs values were selected to match image noise, not dose. BHCT *CTDI*_*vol*_ values ranged from 1.3 to 7.9 mGy; hence, the lowest dose BHCT scans (1.3 mGy) were acquired with six times less dose than the highest dose BHCT scans (7.9 mGy).

#### 2.1.4 WMS scans

In order to evaluate how image noise affects CT-ventilation biomarkers, CT scans were acquired in live WMS subjects. Schomberg et al. (2016) has detailed the anatomical and physiological similarities between this specific porcine breed and human subjects, including strong respiratory system similarities. For the present study, WMS in early adulthood were used with weight and lung size matching that of human adults. Subject weights ranged from 70 to 100 kg. Our group has previously used WMS for pre-clinical studies (Wallat et al., 2021; Wuschner et al., 2021; Wuschner et al., 2022a; Wuschner et al., 2022b).

Following phantom imaging, five WMS subjects were each imaged on two separate scan dates with breathing precisely controlled by mechanical ventilators and while under general anesthesia, minimizing in-scan subject motion. The animal study was reviewed and approved by the Institutional Animal Care and Use Committee (IACUC). Supplementary Material provides details of WMS subject management throughout the imaging study; drug and anesthesia administration methods were approved by the American Veterinary Medical Association (AVMA).

**FIGURE 2:**
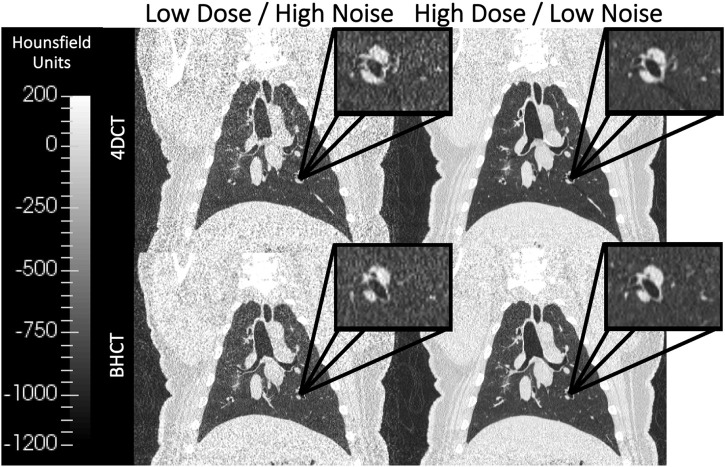
Coronal images of scans acquired at multiple dose levels are shown. The top row shows 4DCTs acquired with low dose (CTDI_*vol*_ = 6.07 mGy) on the left and high dose (CTDI_*vol*_ = 60.7 mGy) on the right. Similarly, the bottom row shows 4DCTs acquired with low dose (CTDI_*vol*_ = 1.3 mGy) on the left and high dose (CTDI_*vol*_ = 7.9 mGy) on the right. Qualitatively, the lower dose images in the left column show increased image noise relative to the high dose images of the right column. All four displayed images were reconstructed without IR applied.

For all subjects on each of the two scan dates, repeated 4DCT and BHCT scans with different levels of image noise were acquired of each subject. Two consecutive 4DCTs were acquired; the first 4DCT was acquired using 100 mAs and the second 4DCT was acquired with 10 mAs, as shown in the top row of Figure 2. During 4DCT acquisitions, subjects were ventilated at 15 breaths per minute (BPM) with a 1,000 cubic centimeter (cc) tidal volume (TV). The Varian Real-Time Position Management (RPM) system (Varian Medical Systems, Inc., Palo Alto, CA) was used to track subjects’ chest positions during 4DCT acquisitions. RPM respiratory traces were used to reconstruct 4DCT image data into 10 breathing phases classified by their inspiratory (IN) or expiratory (EX) percentage (0EX, 20IN, 40IN, 60IN, 80IN, 100IN, 80EX, 60EX, 40EX, 20EX), as previously described (Han et al., 2011).

**FIGURE 3:**
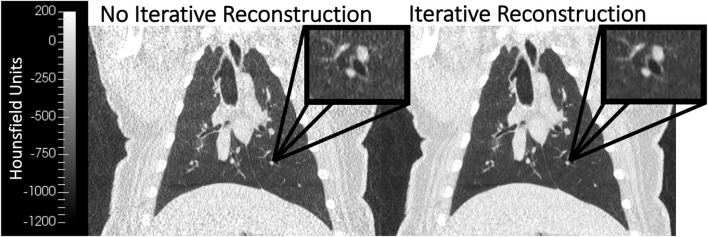
Coronal WMS images reconstructed without (left) and with (right) iterative reconstruction (IR) are shown. The image with IR applied demonstrates noise reduction when compared to the image without IR. Both images were acquired with the same image acquisition technique (10 mAs 4DCT).

BHCTs were also acquired on each scan date at 10 intermediate mAs levels (ranging from equivalent noise to 15–100 mAs 4DCTs). Coronal BHCT images acquired with highest (equivalent to 15 mAs_4*D*_) and lowest (equivalent to 100 mAs_4*D*_) noise levels are shown in the bottom row of Figure 2. BHCT scans were acquired at three distinct lung volumes: maximum expiration (MEBH), maximum inspiration (MIBH) and 80% inspiration (80%Insp) which are analogous to 4DCT 0EX, 100IN and 80IN, respectively. The two different inspiratory volumes (80%Insp and MIBH) were imaged to allow multiple TV options to facilitate equivalent TV (ETV) matching since Jacobian ventilation values are volume dependent (Du et al., 2013). For each of the three BH volumes, the subject held constant pressure to maintain the volume while alternating craniocaudal and caudocranial scans were acquired with decreasing mAs values. This BHCT image acquisition method has been previously described in detail (Flakus et al., 2020). Scans were always acquired in order from highest to lowest dose. All scans were reconstructed with the same parameters used for phantom scans, including both with and without SAFIRE3 IR. Figure 3 shows an example 10 mAs 4DCT reconstructed with and without IR applied for noise reduction.

### 2.2 CT-ventilation calculation

CT-Ventilation maps were calculated using the Jacobian determinant of the transformation between inhale and exhale volumes to estimate local tissue expansion as a surrogate for ventilation, as previously described (Reinhardt et al., 2008). The Jacobian-based technique uses image registration between inhale and exhale lung volume images to calculate tissue expansion. Regions of high expansion indicate regions of increased ventilation. Prior to calculating Jacobian values, a deep-learning lung masking segmentation algorithm was used to mask lung volumes (Hofmanninger et al., 2020). Next, all BH and 4DCT breathing phase volumes were calculated from the lung masks. TV were calculated as the difference between inhale volumes and the end exhale volume (MEBH or 0EX) and were used to select ETV between compared scans for effort correction.

When a subject receives consecutive 4DCT scans, their TV may differ, which affects derived CT-ventilation biomarkers. In order to account for this volume effect, effort correction is implemented by selecting inhale phases with the closest matching TV (also referred to as equivalent TV—ETV) between compared scans. As an example, if a subject receives two consecutive 4DCTs and has a much larger full inhale volume on the second scan, the overall TV (i.e., 100IN-0EX) will not match between the two scans. By excluding the full inhale volume of the second scan, the new TV (80IN-0EX) may more closely match the TV of the first scan and be used for ensuing calculation of CT-ventilation biomarkers. Selecting inhale volumes to achieve ETV between scans accommodates for differences in breathing effort that affect lung volumes; this method of effort correction has previously been described in depth (Wallat et al., 2020).

Du et al. (2013) has previously highlighted the importance of effort correction when comparing volume-dependent Jacobian values. Registration of expiratory and ETV-selected inspiratory volumes was achieved using a B-spline deformable image registration with a sum of squared tissue volume differences (SSTVD) metric to account for lung density changes between different volumes (Cao et al., 2012).

For BHCT acquisitions, the ETV-selected inspiratory image (80%Insp or MIBH) was registered to the MEBH image. The Jacobian determinant was calculated directly from the corresponding transformation matrix. Each voxel-level Jacobian value estimates local lung tissue expansion between exhale and inhale BH volumes as a surrogate for ventilation.

For 4DCT acquisitions, the LER-N Jacobian-based ventilation calculation method, initially introduced by Shao et al. (2020), was used to generate ventilation maps. This method involves registering multiple breathing phases included in the ETV to the full expiration phase (0EX). Jacobian determinants are then calculated from all the registrations and combined to determine maximal expansion throughout the breathing cycle. Using image data from more than two breathing phases in LER-N accounts for out-of-phase regions that do not expand and contract in conjunction with the global lung volume. Previous work has reported that 7.6% of WMS subjects’ lung volume is out-of-phase on average (Flakus et al., 2020).

Ventilation maps were generated from scans acquired at all noise levels with and without IR applied. Therefore, 24 CT-ventilation maps were generated per subject per scan date; four 4DCT ventilation maps included two image noise levels each with and without IR and 20 BHCT ventilation maps included 10 image noise levels each with and without IR.

### 2.3 Quantitative comparison

Comparison of CT-ventilation derived from scans acquired with differing image noise levels was facilitated through deformable image registration (Cao et al., 2012). For each subject, ventilation maps were only compared between those with the same acquisition type (BHCT or 4DCT) acquired on the same day. All reduced mAs (increased noise/decreased dose) scans were registered to the highest mAs (decreased noise/increased dose) scans in order to compare CT-ventilation maps in the reference frame of the higher mAs scan. Acquisitions with and without IR were compared separately.

Similarity between CT-ventilation biomarkers with different amounts of image noise was quantified using metrics of gamma pass rate (Γ), Spearman correlation coefficient (*ρ*) and coefficient of variation (CoV_*JR*_) of the Jacobian ratio (JR). Higher Γ and *ρ* values correspond to better consistency between compared CT-ventilation; on the contrary, lower *CoV*_*JR*_ values correspond to better consistency. Eqs 6, 8 define two of the three metrics for comparing CT-ventilation values from high and low mAs Jacobians *J*_1_ and *J*_2_, respectively. Γ, defined in Eq. 6 (Low et al., 1998), was evaluated locally with 2 mm distance-to-agreement (DTA) and 6% Jacobian intensity (JI) criterion (C) as has been reported previously in evaluation of CT-ventilation biomarkers (Wallat et al., 2020; Flakus et al., 2020) because it is the standard deviation of Jacobian values when evaluated with repeat scans in human subjects. Spearman correlation was calculated at the voxel level and classified as strong if *ρ* ≥ 0.8, following guidelines initially proposed by Zou et al. (2003). Voxel-wise JR is defined in Eq. 7. CoV_*JR*_ is then calculated from the mean (*μ*_*JR*_) and standard deviation (*σ*_*JR*_) of JR, shown in Eq. 8.

$$\gamma \left(x_{1}\right)=\underset{x_{2}}{\text{min}}\left[\sqrt{{\left(\frac{x_{2}-x_{1}}{C_{\mathrm{DTA}}}\right)}^{2}+{\left(\frac{J_{2}\left(x_{2}\right)-J_{1}\left(x_{1}\right)}{C_{JI}/100\%\times J_{1}\left(x_{1}\right)}\right)}^{2}}\right]\;\;\;\;\;\;\;\;\forall x_{2}\left\{\begin{array}{c} \text{passes if}\;\gamma \leq 1\;\\ \text{fails if}\;\gamma >1\; \end{array}\right.$$ (6)

$$JR=\frac{J_{2}}{J_{1}}$$ (7)

$$CoV_{JR}=\frac{\sigma_{JR}}{\mu_{JR}}$$ (8)

Since Γ and *CoV*_*JR*_ directly compare ventilation values, these metrics are sensitive to significant TV differences between compared scans. To best isolate the effect of image noise, any scans with TV differences > 100 cc were excluded from Γ and *CoV*_*JR*_ analysis; this exclusion is consistent with previously reported findings (Reinhardt et al., 2008) regarding volume matching between compared ventilation maps. Since *ρ* only compares the CT-ventilation magnitude and not direct values, no data was excluded from Spearman correlation analysis.

## 3 Results

### 3.1 Image noise relationship to mAs

Based on image noise values calculated from uniform phantom scans, the relationships between image noise and mAs for both 4DCT and BHCT acquisition types are given in Eqs 9, 10. Both fits had *R* ^2^ values greater than 0.99. Substituting numerical values for constants *A*_4*D*_, *B*_4*D*_, *A*_*BH*_ and *B*_*BH*_ fromEqs 9, 10 intoEq. 5 yields Eq. 11 with the numerical relationship between 4DCT and BHCT mAs values that produce the same level of image noise. The right side of Eq. 11 shows an order of magnitude estimate that using approximately 60% of the mAs used in a 4DCT would lead to an equivalent noise level for BHCT scans. This relationship between *mAs*_4*D*_ and *mAs*_*BH*_ is expected due to a nuance of 4DCT acquisition. For the scanner used in this work, 4DCTs were reconstructed from limited CT angle projections (180^*°*^ + fan angle) as opposed to the full 360^*°*^ used for BHCT reconstructions (Rietzel et al., 2005; Han et al., 2011). Since reduced projection angles leads to increased image noise in CT (Gong et al., 2019) and 4DCTs were acquired with fewer projections than BHCT scans, 4DCTs had higher noise levels than BHCTs when using the same mAs values. Therefore, to achieve equal noise, BHCT scans needed lower mAs values than 4DCT scans. The left side of Eq. 11 was used to calculate BHCT mAs values used to image WMS.

$$Noise_{4DCT}=405.4\times {\left(mAs_{4D}\right)}^{-0.487}\;\;\;\;\;\;R^{2}>0.99$$ (9)

$$Noise_{\mathrm{BHCT}}=325.5\times {\left(mAs_{BH}\right)}^{-0.497}\;\;\;\;\;\;R^{2}>0.99$$ (10)

**FIGURE 4:**
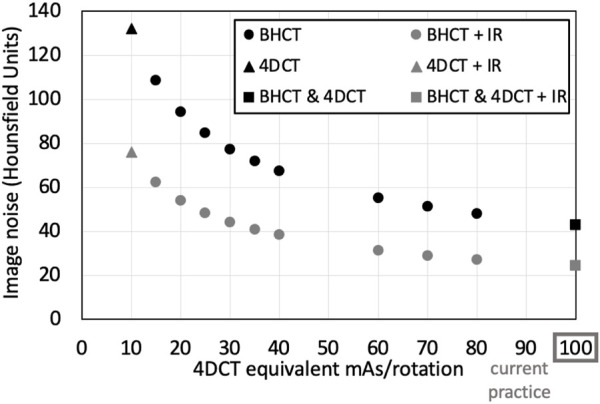
Noise levels of scans acquired in WMS.

The 10 mAs_*BH*_ values used in this work are listed in Table 1 along with mAs_4*D*_ that produce images with the same image noise level when evaluated in a uniform phantom. Noise levels (with and without IR applied) for all scans acquired in WMS subjects are shown in Figure 4. For 4DCT acquisitions without IR, highest and lowest dose acquisitions had image noise values of 133 and 43 HU, respectively. This corresponds to a 209% image noise increase between acquisitions. For BHCTs, highest and lowest noise values were 103 and 43 HU, corresponding to a 142% increase in image noise.

$$mAs_{BH}={\left(0.8\times {\left(mAs_{4D}\right)}^{0.487}\right)}^{\frac{1}{0.497}}\approx 0.6\times mAs_{4D}$$ (11)

**TABLE 1:** Ten mAs_*BH*_ used in this work are listed. mAs_*BH*_ were selected using Eq. 11 to find equivalent noise (with units of HU).

mAs_*BH*_	Equivalent noise [HU] mAs_4*D*_
10	15
12	20
15	25
18	30
21	35
24	40
36	60
41	70
47	80
59	100

### 3.2 Breathing parameter control

**FIGURE 5:**
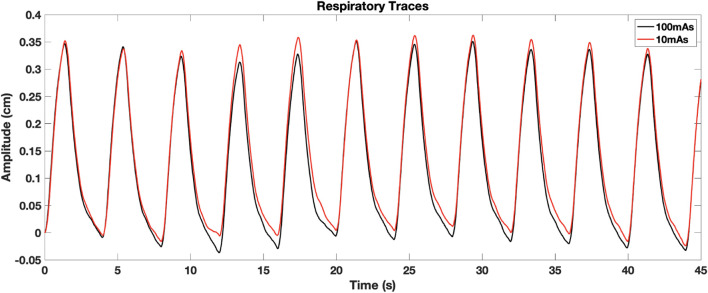
RPM breathing traces for consecutively acquired 4DCTs are shown.

The impact of image noise was studied in mechanically ventilated WMS in part for the ability to tightly control their breathing parameters affecting ventilation. For 4DCT acquisition, TV differences between scans acquired on the same day had mean [range] values of 5 ± 3 [2 13] cc. The 4DCT breathing period had average coefficient of variation of 0.010 ± 0.009 where a smaller value indicates better agreement. Figure 5 shows an example of breathing traces between 10 and 100 mAs 4DCTs, demonstrating a highly reproducible breathing pattern while on mechanical ventilation.

**FIGURE 6:**
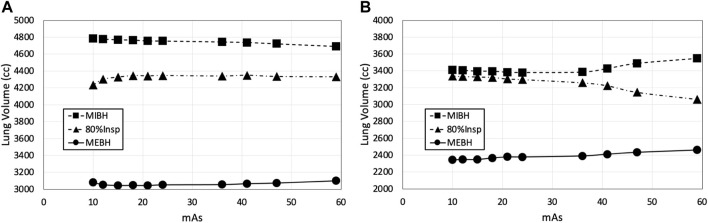
BHCT volume control is shown through two examples. In panel **(A)**, volumes remained relatively consistent across all ten scans of all three volumes. In panel **(B)**, clear volume drift is seen across scans. Scans were always acquired in decreasing 59–10 mAs order, so volume drift typically had a larger impact on comparisons involving low mAs biomarkers.

Breathing parameter control for BHCT scans was dictated by how consistently subjects held pressure at a given volume. Figure 6 shows two examples of BHCT volume control: one with consistent volumes across scans and a second showing volume drifting between acquired scans. The volume drift shown on the right in Figure 6 demonstrates that while uncertainties associated with breathing variability are reduced in WMS imaging, they are not completely eliminated. These examples can be used to contextualize the effect volume differences had on results. For scans on the left plot of Figure 6 had an average gamma pass rate of 91.5% compared to 89.7% for the scans of the right plot. Specifically, scans with TV differences < 100 cc had average Γ of 94.1% but scans with TV differences > 100 cc had average Γ of 87.5%. Since scans were always acquired in order of lowest to highest dose, any instances of volume drift disproportionately affected low mAs CT-ventilation comparison to full mAs values. Due to volume drift, BHCT TV differences were larger than those of 4DCT with averages of 54 ± 32 cc (range: 1–99) when excluding scans with TV difference > 100 cc and 72 ± 51 cc (range: 1–212) without exclusions.

### 3.3 4DCT robustness to image noise

**FIGURE 7:**
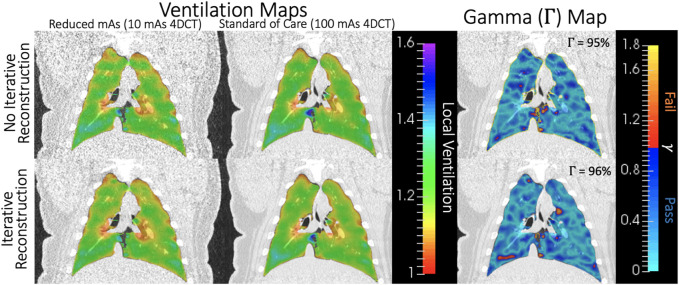
For one subject on one scan date, Jacobian values for all four CT-ventilation maps (10 and 100 mAs with and without IR) are shown in the two leftmost columns. On the right, gamma maps are shown for comparing the first two columns without (top) and with (bottom) IR applied. Blue-shaded voxels passed the Γ analysis, which was 95%–96% of the lung volume in this example.

For one subject on one scan date, all four CT-ventilation maps generated from 4DCT are shown in the first two columns of Figure 7. In these maps, Jacobian values (range 1–1.6) provide an estimation of relative tissue expansion. Values > 1 indicates tissue expansion, which is used as a surrogate for local ventilation in this work. Although non-linear, a Jacobian value of 1.1 roughly corresponds to 10% tissue expansion. Qualitatively, all ventilation maps showed strong similarity, generally identifying the same lung regions as being high or low ventilating. Gamma analysis comparing reduced and standard of care mAs 4DCT biomarkers are shown in the rightmost column of Figure 7. Blue-shaded regions passed the analysis, while regions in warm colors (corresponding to *γ* > 1) failed the analysis. Γ maps for comparisons with and without IR show spatial similarity. Without IR, 95% of the lung volume passed the Γ analysis between low and high dose 4DCTs for this subject. With IR, the pass rate improved to 96%.

**FIGURE 8:**
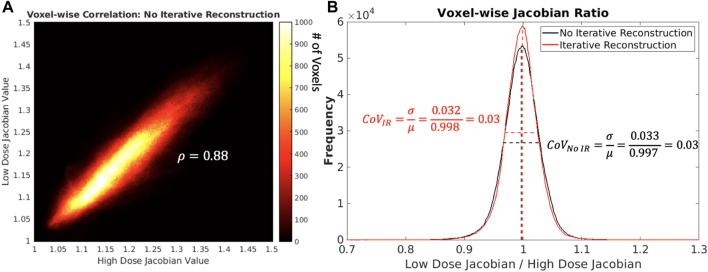
In panel **(A)** on the left shows, a heat map comparing low and high dose 4DCT Jacobian values for one subject on one scan date is shown. The colorbar indicates the number of voxels in each Jacobian value bin. In panel **(B)** on the right shows, voxel-wise Jacobian ratios with and without IR are shown. Mean and full width half maximum values are shown as dotted lines to highlight how *CoV*_*JR*_ was calculated.

For the same subject and scan date shown in Figure 7, visual representations of voxel-wise *ρ* and *CoV*_*JR*_ metrics between low and high dose 4DCTs is shown in Figure 8. In the voxel-wise heat map between high and low dose 4DCT Jacobians, voxels are clustered around the *y* = *x* diagonal. This clustering shows that the majority of voxels directly agree and many very closely agree. Similarly, the voxel-wise Jacobian ratio (JR) distribution in Figure 8 has a mean value of nearly one, which would indicate perfect agreement. JR values primarily range from 0.9 to 1.1, indicating that most high and low dose ventilation values agree within 10% at the voxel-level. The JR distribution with IR applied is slightly tighter than without IR, but did not meaningfully change the *CoV*_*JR*_ value for the subject.

Average and standard deviation of Γ, *ρ* and CoV_*JR*_ values between 10 and 100 mAs 4DCT acquisitions are shown in Table 2. The first column of data quantifies similarity when IR was not applied. The second column lists values when comparing CT-ventilation maps from images with IR applied. The listed *p*-values in the final column are from two-sided student’s t-tests comparing results with and without IR applied. IR did not make a significant difference for any metric (*p* > 0.05), indicating that noise reduction through IR did not increase similarity between derived ventilation maps.

**TABLE 2:** Quantitative metrics for similarity between ventilation maps derived for 4DCT scans with different noise levels are listed. Results with and without IR are listed separately and were compared using a student’s *t*-test and have *p*-values listed in the right most column.

Metric	No iterative reconstruction	Iterative reconstruction	*p*-value
Γ	92.6% ± 2.8%	93.4% ± 4.1%	0.65
*ρ*	0.88 ± 0.03	0.90 ± 0.04	0.12
CoV_*JR*_	0.039 ± 0.009	0.034 ± 0.003	0.13

### 3.4 BHCT robustness to image noise

**FIGURE 9:**
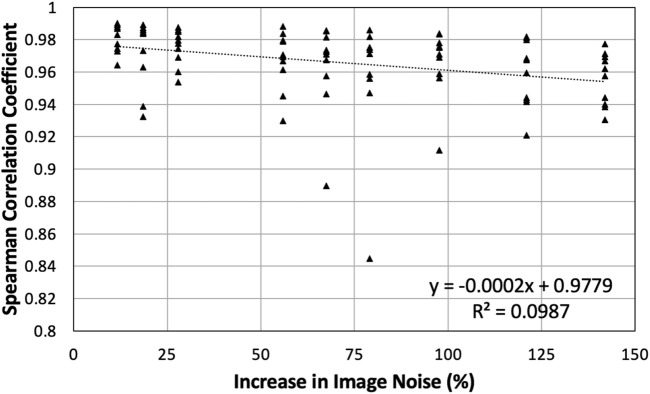
All Spearman correlation coefficients between full and reduced dose BHCT biomarkers without IR are plotted. Points are plotted based on the percentage increase in image noise of the reduced dose scan. Compared to 59 mAs, reduced dose scans of 10–47 mAs had increases in image noise of 12%–142%.

Since BHCT scans were acquired with 10 different mAs values, biomarker comparison entailed comparing nine reduced dose levels (corresponding to 10–47 mAs) to the full dose level (corresponding to 59 mAs). These comparisons were made both with and without IR applied. Figure 9 shows all *ρ* values for BHCT biomarkers between full and reduced dose scans without IR as a function of percent increase in image noise. As demonstrated in the plot, no clear correlation was identified between increased image noise and the Spearman correlation coefficient between full and reduced dose BHCT biomarkers. Summary results combining all reduced dose levels are given in Table 3. Similar to 4DCT results, IR application did not significantly affect results (*p* > 0.05). When comparing 4DCT and BHCT results, Γ and *CoV*_*JR*_ were not significantly different (*p* > 0.05) but *ρ* were (*p* < 0.001).

**TABLE 3:** Summary quantitative metrics comparing nine reduced dose BHCT biomarkers to full dose biomarkers are given. IR application was compared using a student’s *t*-test and did not show a significant difference for any metric (*p* > 0.05).

Metric	No iterative reconstruction	Iterative reconstruction	*p*-value
Γ	92.9% ± 4.3%	92.8% ± 4.4%	0.55
*ρ*	0.97 ± 0.02	0.97 ± 0.03	0.74
CoV_*JR*_	0.034 ± 0.007	0.033 ± 0.006	0.13

## 4 Discussion

### 4.1 WMS model

To our knowledge, this is the first work reporting and quantifying the impact of image noise on CT-ventilation biomarkers. Similar lung size, weight and other physiological attributes between WMS and humans increase the applicability of the results toward clinical implementation. These commonalities suggest that WMS CT-ventilation response to image noise is representative of how CT-ventilation biomarkers derived in humans will be affected. Unlike humans, imaging in WMS allowed reduction of uncertainties like in-scan subject motion and erratic breathing patterns. Figures 5, 6 visually demonstrate small breathing-related uncertainties that remain when imaging WMS, but overall tight control of parameters. WMS also better facilitated the investigation of a wide range of image noise levels due to tolerance of increased CT dose from consecutive scanning.

### 4.2 Robustness to image noise

Quantitative results presented in Tables 2, 3 show overall strong agreement between full and reduced dose biomarkers. Without IR, low and high noise 4DCT scans had corresponding noise values of 43 and 133 HU. When comparing these biomarkers with three times more noise, average Γ and *ρ* were 93% and 0.88. All Spearman correlations satisfied *ρ* > 0.8, indicating strong agreement as detailed by Zou et al. (2003) Our group previously reported CT-ventilation repeatability in WMS with average Γ and *ρ* of 89% and 0.92, respectively (Flakus et al., 2020). Those repeatability values compared consecutively acquired 100 mAs 4DCTs with no parameter changes between scans. Of note, breathing parameters affecting ventilation were more tightly controlled in the present study than the previous repeatability study. In that work, 4DCT breathing period CoV and TV differences were 0.004 and 25 cc on average (Flakus et al., 2020), compared to 0.001 and 5 cc in this work. Similar Γ and *ρ* values from the two studies indicate that image noise is not a driving factor when comparing Jacobian CT-ventilation biomarkers.

BHCT biomarker comparisons between different noise levels also show similar levels of agreement between reduced dose comparisons in the present study (average Γ = 93%, *ρ* = 0.97) and repeatability comparisons in a previous study (average Γ = 83%, *ρ* = 0.97) (Flakus et al., 2020). Increased Γ values in this study are consistent with the improved TV control. BHCT scans were used to evaluate several intermediate image noise levels; from Figure 9, there is not a clear trend with the amount of image noise increase and the agreement between biomarkers. All Spearman values are labelled as strong (*ρ* > 0.8) (Zou et al., 2003), with all but two values above 0.9 and most values above 0.94. With noise levels ranging from 43 to 103 HU these results show the effect of increasing noise by up to almost two and a half times. This consistency across nine image noise levels further supports the finding that the Jacobian-based method used in this work is invariant to HU variations caused by image noise.

The minimal impact of image noise on Jacobian values is further validated by the results of comparing biomarkers with IR applied. Although IR reduced image noise (Figure 3), its application did not significantly change resultant quantitative metrics (Tables 2, 3). Figure 7 also shows how closely biomarkers generated from scans with and without IR agree spatially. Since increasing noise through reducing the mAs did not drastically reduce biomarker agreement, it is consistent that decreasing noise by applying IR did not drastically improve biomarker agreement. Maintaining consistent ventilation values in the presence of increased image noise highlights overall robustness of Jacobian-based CT-ventilation biomarkers.

### 4.3 Dose reduction

Since image noise and dose are inversely related, tolerance of increased image noise indicates a potential for dose reduction. In this work, biomarkers from standard of care 4DCTs were compared to biomarkers from scans with 10 times less dose (60.7 vs. 6.07 mGy *CTDI*_*vol*_). For BHCTs, doses ranged from 1.3 to 7.9 mGy *CTDI*_*vol*_, allowing for up to six times dose reduction between the highest and lowest dose scans. 4DCTs require higher doses than BHCTs but the value of 4DCT versus BHCT based ventilation is application dependent. Whether acquiring 4DCT or BHCT scans, substantial dose reduction can be realized without sacrificing CT-ventilation quality when using the Jacobian determinant computed directly from the deformable image registration transformation matrix.

### 4.4 Jacobian-based CT-ventilation

This work focused on a single CT-ventilation post-processing technique, namely computing the Jacobian determinant directly from deformable image registration transformation matrices between inhale and exhale volumes. The robustness to image noise presented here is an advantage of Jacobian-based biomarkers specifically, and does not necessarily apply to other CT-ventilation derivation methods. For example, the commonly used technique of estimating ventilation directly from HU would likely become less reproducible in the presence of increased HU variation.

As a common alternative to the Jacobian-based method presented in this work, HU-based CT-ventilation calculation assumes that HU change between inspiratory and expiratory images is solely due to the addition of air, changing the HU value as air is inhaled throughout the breathing cycle. Regional ventilation is calculated using the difference between voxel-wise inhale and exhale HU values (Kipritidis et al., 2016). The uncertainty in the CT-ventilation value for this method is therefore dependent on the uncertainty of HU values. Based on the standard error propagation formula, the variance of HU-based CT-ventilation is directly proportional to the variance of HU values. Images with increased noise have a larger HU standard deviation (and therefore variance). Figure 4 shows the standard deviation of HU and how it increases in high noise (and correspondingly low dose) scans. When calculating CT-ventilation using established HU methods, increased image noise would then lead directly to increased uncertainty of derived ventilation biomarkers. Based on the results of this work, invariance to image noise is one advantage that Jacobian-based biomarkers have over those that are directly HU-based. For other CT-ventilation calculation methods beyond using Jacobian determinant or HU-based methods, experiments would need to be performed to quantify image noise dependence.

Since Jacobian-based CT-ventilation can be calculated from 4DCT and BHCT acquisitions, both were evaluated in this work. Both acquisition methods showed similar quantitative agreement between high and low noise based biomarkers. From Tables 2, 3, Γ and *CoV*_*JR*_ agree and were not statistically different while *ρ* was higher for BHCT than 4DCT biomarkers and showed a significant difference. One of three metrics shows that BHCT biomarkers may have stronger agreement in the presence of increased noise; however, both acquisition types showed agreement on par with baseline biomarker repeatability. Whether derived from 4DCT or BHCT scans, Jacobian-based CT-ventilation is advantageously robust to image noise.

### 4.5 Potential clinical applications

This work identified robustness to image noise as an advantage of Jacobian-based CT-ventilation biomarkers; further consideration is needed to determine how this advantage can be leveraged to be clinically impactful. One way of utilizing this advantage would be to reduce the necessary dose for deriving CT-ventilation biomarkers by acquiring reduced mAs CT scans. In current CT-ventilation uses, this dose reduction would mitigate patient risk associated with CT imaging dose. Additionally, dose reduction may allow CT-ventilation usage in more applications. As a viable low dose option for providing spatial distribution of patients’ ventilation, they may be valuable compared to standard of care pulmonary function tests (PFTs); while PFTs have no associated dose, they are only a global measure and have high variance. Diagnostically, spatial ventilation information is relevant in evaluating conditions such as chronic obstructive pulmonary disease (COPD) and emphysema.

For RT applications, reduced imaging dose is minimally meaningful relative to doses delivered during RT. However, the results presented in this work still have potential therapeutic applications. For example, using CT-ventilation to quantify radiation-induced lung damage is an active research area. Acquiring repeated low dose 4DCTs may be advantageous for this application since artifact-ridden 4DCTs often impede the ability to evaluate ventilation changes in response to treatment and/or cause patients to be excluded from clinical studies. Multiple low dose acquisitions can also be valuable for improved characterization of ventilation variance. Further consideration is needed to identify more clinical situations in which the noise invariance advantage of Jacobian-based biomarkers can be best leveraged.

## Data Availability

The raw data supporting the conclusion of this article will be made available by the authors, without undue reservation.
