# Supplementary material for: Quantifying robustness of CT-ventilation biomarkers to image noise
Source: Front Physiol. 2023 Feb 14;14:1040028. doi: 10.3389/fphys.2023.1040028 (PMC9971492; doi:10.3389/fphys.2023.1040028)
Supplement: Supplementary file 1 [file DataSheet1.PDF]

Supplementary Material

1 SUPPLEMENTARY DATA

Five WMS subjects received CT imaging on two dates at the University of Wisconsin-Madison (UW-Madison) in a pre-clinical study approved by the Institutional Animal Care and Use Committee (IACUC). All subjects were in early adulthood and had weights ranging from 70-100 kilograms. Subjects were maintained in a pathogen-free facility by the Biomedical Research Model Services group at UW-Madison between imaging sessions. Initially, Dantrolene, an injectable anesthesia cocktail (Telazol+Xylazine) and gas anesthesia with isoflurane were administered prior to placement of an indwelling venous catheter; this process is detailed in Table S1. Before each imaging session, these drugs were also administered along with a 10 mL saline flush and the subject was intubated.

Throughout each imaging session, vitals were monitored and breathing rates were adjusted to maintain proper SpO2 and ETCO2 levels. When transported, subjects were covered in warm linens and ventilated at 8 breaths per minute (BPM) and 1000 cubic centimeters (cc) per breath. For each imaging session, subjects were taken to the CT suite to be imaged with various breathing maneuvers as described in the WMS Scans section. Following imaging completion, subjects returned to the housing facility where mechanical ventilation and anesthesia were discontinued, and the subject was extubated. This procedure was repeated for a total of two imaging dates, approximately three months apart.

2 SUPPLEMENTARY TABLES AND FIGURES

2.1 Tables

Initial Indwelling Catheter Placement	
Administer:	Dantrolene (5.9 mg/kg)
	Injectable anesthesia cocktail [Telazol (3.0 mg/kg) + Xylazine (1.5 mg/kg)]
	Gas anesthesia with isoflurane at 2%
Percutaneously place catheter on left side of the vena cava	
Collect 20 mL of blood for serum and plasma samples	
Administer 30 mL saline flush	
Discontinue anesthesia	
Fit subject with catheter jacket	

Table S1. Process for indwelling catheter placement prior to swine imaging.
